# Training for departure? Policy, professional portability, and the fragile future of Nepal's nursing workforce

**DOI:** 10.3389/frhs.2026.1851983

**Published:** 2026-05-19

**Authors:** Animesh Ghimire

**Affiliations:** 1 Sustainable Environment Foundation Nepal, Kathmandu, Nepal; 2 School of Nursing, Chitwan Medical College, Chitwan, Nepal; 3 School of Public Health, Chitwan Medical College, Chitwan, Nepal

**Keywords:** aspiration, governance, health equity, ICN, mobility, MoUs, nurse migration, nursing students

## Abstract

Workforce migration is often cast as a matter of personal choice or national shortage. This policy and practice review contends that the prevailing framing inadequately captures the dynamics of nursing mobility throughout the workforce lifecycle. It emphasises that this mobility is influenced by various stages, including education, early professional socialisation, active practice, and international migration. These stages ultimately contribute to systemic challenges by resulting in structural losses that affect the overall workforce. Using Nepal as a case study with wider relevance for low- and middle-income countries, the review examines how *aspiration*, *governance*, and *depletion* become mutually reinforcing rather than discrete phenomena. Drawing on a targeted review of empirical literature indexed in MEDLINE, CINAHL, and Scopus, together with key governmental reports and International Council of Nurses and World Health Organisation documents, and guided by Walt and Gilson's policy triangle, the paper analyses how migration intent is *formed*, *mediated*, and *reproduced* over time. The analysis shows that migration is seeded early, during undergraduate training, where educational-clinical disjuncture, hierarchy, weak professional recognition, and normalised expectations of overseas opportunity begin to orient students toward exit. These aspirations are then structured through governance mechanisms, including bilateral recruitment arrangements, such as the Nepal–UK Memorandum of Understanding, and definitional shifts in nursing that increase nursing's portability and international legibility. The effects are not confined to workforce numbers. They surface in compromised clinical supervision, ethical dissonance, and psychological strain among students; in moral distress, managerial overload, and widening rural disadvantage among practising nurses; and in a broader weakening of the domestic system's capacity to reproduce its own workforce. The review concludes that nurse migration is not simply a retention problem. It is a governance failure in the social and professional reproduction of nursing. Effective policy must therefore move beyond moral appeals to stay and instead align domestic professional revaluation, student-pipeline protection, and reciprocal mobility governance.

## 1 Introduction: recasting the problem

The issue of brain drain in nursing is frequently framed as a mere conflict between individual ambition and national shortages. That framing is now analytically inadequate. Although the global nursing workforce has expanded, the WHO still projects a shortfall of 4.5 million nurses by 2030, with the heaviest gaps concentrated in Africa and South-East Asia, while one in eight nurses now practices outside the country where they were trained (1, 2). Recent policy scholarship shows that contemporary health-worker mobility is no longer adequately explained by wage differentials or personal aspiration alone; it is increasingly shaped by policy implementation failures, destination-country underinvestment in domestic workforce development, and bilateral or multilateral governance arrangements that can normalise “ethical recruitment” while obscuring relations of extraction (3, 4). Yet the effects of migration are not uniform across all source countries: in some settings, emigration coexists with unemployment, overproduction, or rural–urban maldistribution, cautioning against any automatic equation between out-migration and systemic collapse (5). The real question, then, is not whether nurse migration is inherently harmful, but how particular policy configurations distribute opportunity, risk, and loss. This broader trend is becoming increasingly evident across South Asia. Research on India–UK nurse migration indicates that international mobility can be strategically cultivated through commercial training pathways and intermediary actors. Additionally, studies on Indian nurses migrating abroad have highlighted how limited local opportunities and segmented labour markets contribute to the normalisation of overseas practice as a typical professional aspiration (6, 7).

The inquiry into the ethical implications of health workforce recruitment is particularly salient in Nepal. This issue transcends a narrow migration debate; it represents a pressing crisis of national planning. While Nepal's Human Resources for Health (HRH) Strategy 2021–2030 (8) and the Health Sector Strategic Plan 2023–2030 (9) explicitly aim to strengthen workforce development and utilisation, the structural reality remains starkly disconnected from these goals. The most recent WHO country profile identifies international migration, uneven distribution, and chronic public-sector vacancies as major, unresolved challenges (10). This contradiction is most sharply illustrated by current labour-market data. Although Nepal produces a robust pipeline of approximately 7,610 nurses and midwives annually, only 77.96% of sanctioned public-sector nursing posts are actually filled (10, 11). This glaring misalignment among production, deployment, and domestic absorption serves as a primary structural driver of outward mobility. Out-migration has become a salient characteristic of the nursing profession in Nepal. Current estimates indicate a nurse-to-population ratio of 4.1 per 1,000 individuals (12), resulting in fewer than 83,966 registered nurses for a population exceeding 30 million (13). This statistic highlights the acute severity of the healthcare staffing crisis within the country. Furthermore, this outward pressure is actively institutionalised: despite Nepal's presence on the WHO's health workforce safeguards list, the 2022 Nepal–UK Memorandum of Understanding (MoU) formally established a government-to-government recruitment pathway, further embedding international exit into the domestic nursing trajectory. This initiative is situated within a policy framework that the WHO's code of practice would typically advise against, thereby raising concerns about compliance with international guidelines aimed at ensuring the ethical recruitment of health professionals (14, 15). At the same time, the International Council of Nurses' (ICN's) 2025 renewal of the definitions of “nursing” and “a nurse” has elevated the stakes of professional regulation by separating the discipline from the regulated practitioner and reasserting nursing as a science-based, rights-grounded, leadership-capable profession (16). For LMICs, however, that professional elevation is double-edged: it can enhance domestic status, regulatory frameworks, and influence, while concurrently augmenting the visibility and transferability of a workforce already characterised by high global mobility (17).

What makes Nepal analytically significant is that this process can now be traced across the full professional pipeline. Emerging evidence shows that migration intentions are formed during undergraduate education (18), that some of those intentions are enacted within a year of graduation (19), and that the resulting shortages reverberate through clinical learning (20), ethical formation (21), student well-being (22), and nurse management (23). This article argues that the Nepalese nurse brain drain is best understood not as an aggregate of personal choices, but as a policy-mediated cycle in which student *aspirations*, *bilateral recruitment*, and transnational *professional standards* interact to reorganise who leaves, who stays, and who bears the costs. Nepal is therefore not a marginal case. It is a strategically revealing example of a wider LMIC dilemma: how to professionalise nursing, enable fair mobility, and still reproduce a viable domestic workforce.

## 2 Analytic framework and approach

Policy analysis, as articulated by Browne and colleagues, is significant for its critical examination of the processes through which authorities define problems, prioritise specific stakeholders, and legitimise particular solutions (24). This approach underscores the complex interplay between power dynamics and policy formulation, revealing how choices are influenced by the framing of issues and the interests foregrounded in the policymaking process. The primary framework utilised in this paper is Walt and Gilson's health policy triangle. Its enduring significance lies in addressing the persistent tendency in health reform debates to concentrate on policy content while overlooking the interrelated roles of context, process, and actors. Subsequent methodological research has underscored the importance of employing theoretically explicit and reflexive policy analysis, particularly in LMICs, where the political implications of implementation are often as crucial as the design itself (25, 26). To ensure the framework is applied systematically, this paper explicitly operationalises each component of the triangle. In this analysis, content refers to the formal policy instruments and normative texts under examination, including the Nepal–UK MoU (27), the WHO ethical recruitment code (15, 28), relevant national HRH policy direction (8, 9), and the ICN's renewed definitions of “nursing” and “a nurse” (17). Context refers to the political economy within which these instruments operate: Nepal's workforce production–absorption mismatch, rural–urban maldistribution, gendered professional hierarchies, historical UK–Nepal labour relations, and the wider constraints common to LMIC health systems. Actors include undergraduate nursing students, practising nurses, nurse managers, educators, regulators, ministries, UK employers, and transnational bodies such as the WHO and ICN. Process refers not only to formal policy formulation, but also to negotiation, implementation, translation, adaptation, and feedback effects over time. Recent reviews of the health policy triangle show that its explanatory strength depends precisely on this kind of explicit specification of how the four domains interact in practice (29).

Importantly, the policy triangle is not applied here as a closed system. Recent scholarship cautions against relying on a single model for complex health policy problems, noting a persistent disciplinary bias toward agenda-setting at the expense of implementation analysis (30). To bridge this gap, the triangle is integrated with an implementation-science perspective. Specifically, Nilsen's taxonomy provides a valuable framework by distinguishing between implementation processes and the underlying determinants of success or failure. This approach shifts the focus beyond formal policy adoption to emphasise practical barriers, sequencing, and local adaptation (31). This addition is especially important in LMIC settings, where policy effects are often mediated by organisational discretion, uneven capacity, and local power relations rather than by formal policy intent alone (32).

This focus on implementation is crucial because the object of analysis involves both a bilateral recruitment arrangement and a transnational professional norm. For example, the ICN's 2025 report conceptually separates “nursing” from the individual “nurse,” grounding the profession in science, social justice, and regulation while highlighting professional mobility as a key implication (16). Emerging scholarship suggests these renewed definitions strengthen nursing's policy authority, but they also raise critical questions about how such norms translate across unequal health systems (17, 33). Consequently, this article utilises the Nepalese context as a cumulative case archive to address two core questions: how is transnational mobility politically structured within a domestic health system, and how are global professional norms translated, contested, or adapted within that local reality?.

## 3 The making of migration intent during training

Migration intent among nursing students is a developmental trajectory, not a discrete choice made upon graduation. As recent reviews of health-worker mobility in LMICs illustrate, this intent is assembled through intersecting macro-, meso-, and micro-level pressures. Labour-market conditions, professional status, household obligations, insecurity, and imagined futures abroad are not sequential considerations; they are simultaneous drivers (34). Comparative studies have documented migration intent among nursing students in Uganda, Nepal, and Ghana, indicating that anticipatory exit is often formed well before professional registration (35–37). This temporal shift is important as it suggests that migration should not be treated as a late-career reaction to frustration, but as an orientation nurtured during professional socialisation itself.

International evidence also challenges the assumption that a stronger commitment to nursing necessarily anchors students to local systems. A recent modelling study found that professional identity and financial incentives both exerted direct effects on nursing students' migration intent, while perceptions of future opportunity shaped the wider decision environment (38). In other words, students may consider exiting the nursing profession not due to a lack of commitment, but rather because they take the profession seriously and aspire to practice in environments that recognise and reward expertise, autonomy, and opportunities for professional growth. Furthermore, it is important to note that intent is not static; it can change as perceptions and experiences within the profession evolve. Evidence from Romania following Brexit and the COVID-19 pandemic showed that major geopolitical and public health events altered nursing students' willingness to seek employment abroad, underscoring that the desire to migrate is policy-sensitive and socially contingent rather than a stable personality trait (39).

The pandemic and its aftermath sharpened this recalibration. A recent scoping review of undergraduate nursing students' (UNSs) motivations found that career decisions are now shaped by an expanded moral-material calculus spanning personal, organisational, financial, political, environmental, and gender-related domains (40). A second review focused specifically on migration intention similarly showed that economic aspiration, professional fulfilment, anticipated quality of life, family considerations, and educational and policy environments all interact, while the pandemic intensified awareness of occupational risk, health-system fragility, and global inequality (41). The analytic implication is clear: students are not merely “pulled” by better pay abroad. They are learning, during training, how crisis exposure and institutional precarity reorder what counts as a liveable professional future.

In Nepal, this broader pattern acquires sharper specificity. Qualitative research with final-year UNSs shows that migration intent is produced through repeated encounters with an educational-clinical mismatch: students are taught autonomy, evidence-informed judgment, and professional standards, then enter workplaces where nursing knowledge is underused, hierarchy is rigid, wages are markedly unequal, and insecurity is normalised (18). Recent work from similarly resource-limited health systems suggests that idealised images of nursing—often shaped by family, media, and aspiration—can rapidly erode when students encounter structurally constrained practice environments that make competence difficult to exercise and recognition difficult to attain (42). The issue, then, is not simple disillusionment. It is anticipatory professional sorting: students begin to infer, while still in training, that specialisation, respect, and dignified practice may be more attainable elsewhere.

Migration intent is also socially rehearsed. Earlier mixed-methods research in Nepal found that many students decide early in their studies that migration is likely, with postgraduate education abroad emerging as a primary route (37). More recent longitudinal work suggests that, by graduation, migration can already function as a cohort norm—a taken-for-granted next step circulated through peers, families, and stories of successful mobility (19). In the Nepali accounts, this normalisation is also gendered: overseas nursing is imagined not only as employment, but as a route through which women can claim professional authority, family provision, and transnational social standing (18). Once mobility becomes embedded in the ordinary social script of nursing education, retention is no longer a matter of persuading isolated individuals to stay. The years of training themselves serve as the foundational landscape that transforms the prospect of exit into a conceivable, logical, and progressively accepted practice.

## 4 Policy mediation of mobility: when aspiration becomes structured exit

Once the desire to migrate has formed, it does not move nurses across borders unaided. It is translated into actual mobility through an institutional chain of permissions, classifications, pathways, and protections that determines who may be recruited, by whom, and under what conditions. In Nepal, the challenges associated with health workforce sustainability are particularly pronounced. The country is included on the WHO Health Workforce Support and Safeguards List, which highlights nations experiencing acute shortages in health personnel. This designation serves as a cautionary indicator for international recruitment efforts, emphasising the need for careful consideration in sourcing health professionals from Nepal (15). The UK's existing “Code of Practice” enforces this principle by banning active recruitment from “red list” countries, unless there is a formal government-to-government agreement in place. In such cases, recruitment may only occur in accordance with the terms of that agreement, and the country in question will be reclassified to the “amber” list (43). The 2022 Nepal–UK MoU is therefore best understood not as a neutral administrative arrangement, but as an exception-making instrument: it converts a country that would ordinarily be shielded from active recruitment into one that becomes selectively recruitable.

The architecture of the MoU makes this asymmetry hard to ignore. Formally, the text designates implementing agencies on both sides, prohibits charging recruitment fees to Nepali health professionals, and establishes both a local implementation unit and a joint technical committee to oversee cooperation (14). It also notes that the UK will “endeavour” to support Nepal in expanding its healthcare workforce through a mutually agreeable action plan. However, the legal-political weight of the agreement lies in its operationalisation: forging a concrete recruitment pathway, authorising state-led execution, and opening a pilot phase designed to rapidly extract and deploy nursing labour for the National Health Service (NHS) and its partners (44). What is binding, in other words, is the recruitment process; what is softer and more aspirational is the promise of reciprocal capacity building. Yet the actual flow through this pathway remains far less transparent than the architecture implies. To date, neither the Nepali nor the UK government has published a consolidated public dataset specifying how many nurses have ultimately migrated through the MoU route. The clearest publicly disclosed implementation figure comes from Hampshire Hospitals National Health Service (NHS) Foundation Trust, the sole NHS trust named in the pilot phase, which reported in March 2024 that it would welcome 42 trained nurses from Nepal (45). As of March 2025, NHS employers still described the Nepal pathway as an ongoing pilot restricted to Hampshire Hospitals NHS Foundation Trust and its partners, suggesting that the agreement's institutional architecture remains wider than its publicly disclosed realised flow (43). In this sense, the MoU has so far functioned as a tightly contained pilot rather than a demonstrably large-volume recruitment stream.

That imbalance is not unique to Nepal. Comparative governance research on skilled health-worker migration shows that bilateral agreements can sometimes expand training opportunities or facilitate skills exchange, but source-country gains are frequently weakly specified, return migration is often not achieved, and knowledge acquired abroad may not transfer back into resource-constrained practice environments (46, 47). Recent work on health-worker migration governance has therefore argued that the most meaningful safeguard is not rhetorical reciprocity but transparent workforce data, open scrutiny, and a credible strategy for destination-country self-sufficiency (48). On this point, official UK documents are revealing. The NHS “Long Term Workforce Plan” explicitly models future nursing supply in relation to how quickly international recruitment can be reduced, and states that ethical international recruitment of nurses will need to remain at least around current levels in the coming years while domestic expansion catches up (49). NHS England likewise describes overseas recruitment as an important part of the workforce supply strategy and a means of supporting hospitals in accessing the international market for nurses (50). At the same time, external scrutiny suggests that Nepali nurses form part of a much larger influx from countries facing severe workforce shortages; the publicly available UK data do not clearly distinguish between nurses arriving through the MoU pilot and those entering through other migration routes, such as direct application or pre-existing educational and employment channels (51). The implication is clear: the MoU is not peripheral to UK workforce policy. It is one of the prime elements in a broader strategy of managing immediate shortage through externally sourced labour while longer-term domestic reform remains in progress. However, the mismatch between a highly formalised bilateral agreement and an opaque implementation base also means that the “fit” between design and flow cannot yet be fully evaluated—a limitation that is itself policy-relevant, because opacity weakens scrutiny of whether reciprocal commitments are keeping pace with recruitment outcomes.

At the same time, reducing the MoU to extraction alone would miss an important part of its appeal. Empirical work with Nepali nurses shows that some experienced the agreement as long-overdue recognition of their qualifications and as a potentially fairer route than the older pattern of migrating first as students and only later seeking professional employment (52). This matters analytically because it explains why such agreements can command support even in source countries. They offer direct employment, reduce brokerage abuse, and symbolically validate professional competence. Yet the same nurses also read the agreement through a longer historical memory in which Nepalese labour has repeatedly been mobilised to solve British institutional needs—from Gurkha military service to nursing care—and they questioned whether “partnership” language masked a more familiar asymmetry (27, 52). The ethical ambiguity of the MoU arises from its dual role as a means for recognition and redistribution, serving both as an opportunity and externalisation.

A second layer of policy mediation now sits above the bilateral agreement. The ICN's 2025 report explicitly separated “nursing” from “a nurse,” defined the latter as a professional who is educated and regulated to practice, and listed workforce planning and professional mobility among the key global implications of the new definitions (16). Standardisation is meant to clarify identity, scope, and accountability, but it also facilitates mutual recognition across borders by making nursing roles more legible to regulators and employers (16). For Nepal, this creates a paradox. The same definitional shift that can *strengthen title protection, regulatory authority, and leadership claims domestically also increases the international portability of Nepali nursing credentials* if adopted without domestic retention safeguards (17). Hence, policy functions not only as a response to migration but also actively shapes it. Bilateral recruitment agreements facilitate the mobility of professionals by granting permission to exit, while standardised global professional definitions streamline the classification, comparison, and recruitment of these individuals.

## 5 Consequences for undergraduate nursing students

One of the least appreciated consequences of nurse brain drain is that it destabilises the educational conditions under which the next cohort is supposed to be formed. UNSs do not encounter workforce depletion as an abstract policy problem; they experience it as the quality of their clinical education. In Nepal, where clinical placements remain the indispensable site for translating theory into practice, a recent study found that staff shortages were not merely reducing convenience or efficiency but altering the very structure of learning, producing “compromised learning,” isolation, improvised workarounds, and dependence on digital tools in place of sustained human supervision (20). This is significant because international literature does not uniformly portray a negative view of clinical placements. A comprehensive review of studies on clinical learning environments, supervision, and nurse education revealed that nursing students generally assess their placements positively. However, it also indicated that the quality of the supervisory relationship and the pedagogical atmosphere are key factors determining whether these placements effectively serve as educational environments (53). The Nepal case is therefore analytically important not because students dislike clinical settings in general, but because the shortage erodes precisely those relational conditions that make practice educative.

The consequence is not only questionable supervision but also a more profound hollowing out of training. Students in Nepal described themselves as “ghosts, silently observing from the sidelines,” hesitant to ask overburdened nurses for guidance and uncertain whether they were receiving the preparation required for safe professional practice (20). What is striking here is the mismatch between formal exposure and substantive learning: students may still accumulate clinical hours, yet the educational value of those hours declines when teaching, feedback, and supervised repetition are displaced by staffing shortages. This resonates with broader work on professional identity formation. An integrative review revealed that clinical supervisors, reflective spaces, predictability, and a sense of safety are essential for the development of nursing students. Role models are not merely an optional enhancement; rather, they serve as a primary mechanism through which students internalise professional judgment, cultivate self-confidence, and develop ethical competence (54). In a resource-constrained system, the loss of nurses translates to a loss of educators, practitioners, interpreters, and custodians of a safe learning environment. This not only leads to inadequate training but also results in a more insidious pedagogical harm: students are introduced to nursing without the assurance of consistent supervision throughout their degree.

That injury extends into the ethical domain. In one phenomenological study, students described a “dissonance of duty” in which responsibility to Nepal, loyalty to patients, care for family, and concern for personal futures could not be easily reconciled (21). They also described an “eroding ideal,” generated by the disjuncture between classroom commitments to advocacy, empathy, and leadership and the realities of sub-optimal care, exhausted staff, and absent mentors (21). This is not unique to Nepal, but the Nepalese evidence sharpens what broader evidence has already suggested. A recent scoping review found that moral distress in UNSs is commonly triggered by relationships with preceptors and teams, encounters with constrained services, and situations in which students perceive the right course of action but feel unable to act (55). When experienced nurses leave, students lose not only clinical supervision but ethical exemplars—people who can demonstrate how to remain competent, compassionate, and professionally intact under pressure. However, students actively engage in their learning, and one of the more striking findings from Nepal is the emergence of compensatory learning mechanisms. Lacking time with preceptors, students seek guidance from physicians, allied health professionals, patients, and increasingly, generative artificial intelligence (Gen AI) (20). Therefore, the Nepali evidence used here illuminates how students respond to supervision gaps, not to claim that Gen AI has already been proven to be an equivalent substitute for expert teaching.

That said, the Nepali study's findings do not stand in isolation. A growing body of scholarship indicates that AI-supported and digitally mediated pedagogies are rapidly entering nursing education, particularly through conversational agents, adaptive learning tools, and AI-enhanced teaching strategies, although the evidence remains stronger for feasibility and user perceptions than for robust learning outcomes (56, 57). More specifically, the claim that UNSs use generative AI tools to enhance learning is supported by evidence indicating that technology-assisted education can improve the accuracy of medical interpretation among novice learners (58). Studies with UNSs have found that learning software and online educational programs improved interpretation competency, while work with novice medical trainees has shown that app-based and mobile learning can increase diagnostic accuracy, even if retention over time remains inconsistent (59, 60). Nonetheless, in Nepal, Gen AI does not emerge as a fashionable educational innovation; it appears as a pedagogic prosthesis for a staffing crisis. That distinction matters because the question is no longer whether Gen AI can enrich learning, but what it means when students need it to substitute for supervision.

However, students actively engage in their learning, as one significant finding from research in Nepal is the emergence of compensatory learning mechanisms. Lacking time with preceptors, students seek guidance from physicians, allied health professionals, patients, and increasingly, generative artificial intelligence (Gen AI) (20). This adaptive creativity demonstrates resilience, but it also signals institutional abandonment. Students used Gen AI and app-based tools to interpret electrocardiograms, generate preliminary intervention options, and fill gaps left by unavailable supervisors, while still insisting that such tools could not replace human mentorship (20). This is a more politically significant finding than it may first appear. Reviews of generative AI in nursing education suggest that such tools may improve satisfaction, flexibility, and certain dimensions of critical thinking, yet evidence on learning outcomes remains underdeveloped, and ethical concerns about bias, transparency, privacy, and overreliance persist (61, 62). In Nepal, gen AI does not emerge as a fashionable educational innovation; it appears as a pedagogic prosthesis for a staffing crisis. That distinction matters because the question is no longer whether gen AI can enrich learning, but what it means when students need it to substitute for supervision.

The psychological consequences are equally significant. A study of UNSs showed that distress is shaped not by individual fragility but by a patterned interaction of silence, hierarchy, shortage, and moral comparison (22). Students described “shrouded voices,” confiding only in trusted peers because formal disclosure might be read as weakness or incompetence; “performing resilience,” suppressing anxiety to appear fit for nursing; and they described resource scarcity and patient poverty as conditions that made their own distress seem unworthy of attention (22). Global evidence confirms that such distress is not trivial. An umbrella review of meta-analytic evidence found an overall prevalence of mental health issues of 27% among nursing students, with sleep disturbance, burnout, anxiety, and depression among the most common problems (63). Yet the Nepalese evidence adds something more specific: it shows how distress becomes institutionally invisible. Students rely on quiet peer ties because formal systems feel unsafe or culturally unavailable. Peer support can indeed reduce stress and anxiety in higher education, especially through mentoring and peer learning, but the evidence base remains mixed and cannot justify replacing institutional support with informal solidarity alone (64).

Taken together, these findings show that brain drain affects UNSs across three inseparable domains: *competence, conscience, and well-being*. It weakens skill formation by undermining supervision; it distorts ethical development by normalising care ideals that cannot be practised; and it privatises psychological suffering by reframing endurance as professionalism. The consequence is a *self-undermining* cohort. A system that loses nurses does not simply lose current staff; it also trains their successors under conditions that make safe learning, moral clarity, and a sense of belonging harder to achieve.

## 6 Consequences for the workforce and health system

If the nursing education revealed how brain drain destabilises the future cohorts, its consequences within the active workforce expose the system-level price of depletion. In Nepal, nurse managers occupy a particularly revealing position because they sit between policy failure and bedside consequence. They are required to maintain staffing levels, uphold standards, absorb patient and family distress, and protect junior staff, often while continuing to provide direct care themselves. A study from Nepal shows how these managers experience brain drain and persistent moral distress: they are forced to triage, provide suboptimal care, and normalise compromises they know are ethically troubling (23). This is consistent with broader evidence that moral distress among nurse leaders is not an individual weakness but a structural response to chronic conflict between professional obligations and constrained organisational conditions (65).

The managerial accounts are especially important because they make visible a form of harm that workforce statistics routinely miss: the conversion of shortage into ethically degraded care. Nurse managers in Nepal describe themselves as “holding together a crumbling system,” compelled to decide which tasks can be deferred, which patients can safely wait, and how far exhausted staff can be stretched before quality or safety gives way (23). International evidence suggests that these are not simply subjective impressions. A systematic review of LMICs found that poor staffing and high patient loads are strongly associated with missed nursing care, which, in turn, is linked to medication errors, hospital-acquired infections, mortality, and lower quality ratings (66). The situation in Nepal should not be viewed as an isolated case of strain, but rather as a clear illustration of a broader phenomenon: the departure of trained nurses from the healthcare system does not merely reduce workforce numbers; it significantly increases clinical risk within the system.

These effects are also socially patterned. The burden of out-migration is not evenly distributed across Nepal's health system, and this is one of the strongest reasons to treat brain drain as a question of justice rather than solely focusing on labour supply. Nurse managers in rural settings describe the exodus of staff as a “silent epidemic” that deepens already severe inequities in access, timeliness, and continuity of care (23). This aligns with broader evidence that Nepal's health system remains marked by workforce shortages, rural–urban disparities, and high out-of-pocket costs, even as overall population health has improved in urban areas (67). The result is not just fewer nurses in absolute terms, but a redistribution of scarcity toward the places and populations least able to absorb it.

At the same time, the workforce consequences cannot be reduced to a moral framework in which those who migrate are pitted against those who stay. Practising nurses themselves often interpret migration through an ethically ambivalent lens. In a study on the Nepal–UK recruitment agreement, Nepali nurses described the possibility of overseas work as both recognition and a dilemma (52). That ambivalence is analytically important. It shows that workforce depletion is not produced by indifference to Nepal, but often by the collision between professional aspiration and the absence of credible domestic conditions under which expertise can flourish. Nor are destination settings uniformly emancipatory, as reviews of internationally educated nurses' transition experiences have documented discrimination, communication barriers, under-recognition of prior skills, and difficulty translating speciality expertise into equivalent roles within host systems (68). The consequence is a *double injustice*: source countries lose experienced nurses, while destination countries fail to utilise their skills.

Seen in this light, the workforce effects of brain drain are both material and moral. Materially, they intensify staffing gaps, missed care, rural disadvantage, and patient vulnerability. Morally, they burden managers and remaining staff with the task of translating structural neglect into everyday rationing, while casting migration as one of the few available routes to professional survival. Brain drain, then, is not simply movement across borders. It is *a redistribution of opportunity outward and of responsibility inward*—onto the managers, nurses, patients, and communities left to inhabit these consequences.

## 7 Conceptual reframing: beyond “stay or leave”

The prevailing moral vocabulary of health-worker migration remains constrained by an inadequate theoretical premise. By framing migration as a stark choice between remaining and departing, the discourse is frequently reduced to a binary contest between individual autonomy and collective obligation. While this dichotomy highlights a genuine ethical tension, it lacks explanatory depth. Contemporary migration theory has largely transcended such simplifications; de Haas's (69) aspirations–capabilities framework, for instance, demonstrates that mobility is neither unencumbered free choice nor sheer structural compulsion, but rather the product of aspirations and capabilities forged within unequal opportunity systems. Global health workforce debates, however, consistently lag behind these theoretical advancements. They continue to oscillate between reductive condemnations of “brain drain” and uncritical celebrations of “brain circulation,” failing to interrogate how professional mobility is structurally facilitated, normalised, and reproduced over time.

Nevertheless, these conventional frameworks capture several undeniable realities. Foremost, they rightfully recognise nurses as rights-bearing agents whose claims to equitable remuneration, occupational safety, and professional advancement are ethically paramount. Furthermore, they acknowledge the tangible dividends of mobility: remittances frequently offer vital social protection and alleviate immediate poverty in source communities (70), while return migration often cultivates substantial human, financial, and social capital, including enhanced clinical competencies and robust transnational networks (71). Finally, these debates rightly assert that bilateral agreements are not intrinsically exploitative; indeed, source-country scholarship has consistently maintained that managed migration can, in principle, be structured to yield mutual benefit (72).

**Figure 1:**
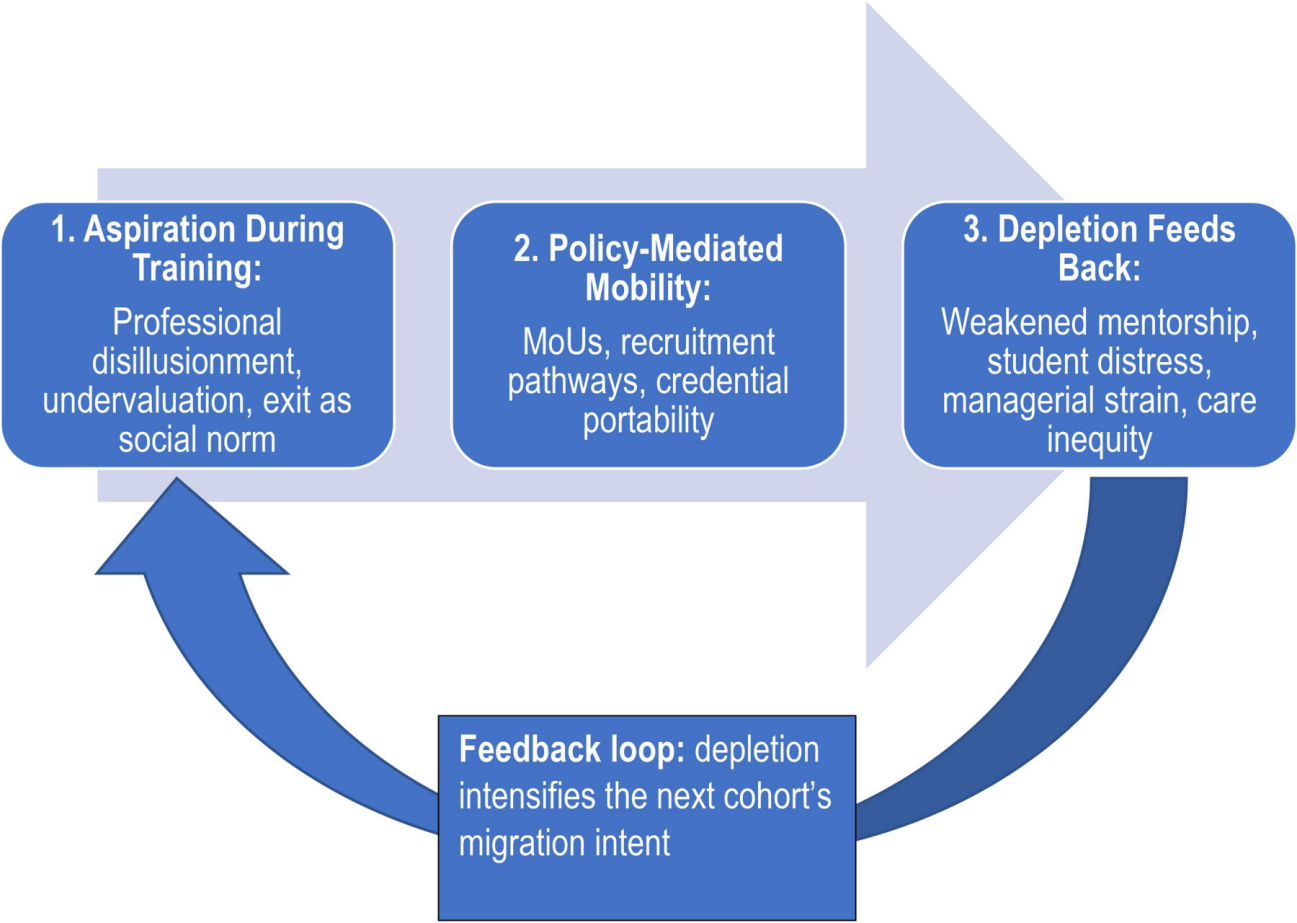
Conceptual diagram of the **aspiration–mediation–depletion** cycle. Migration aspiration is formed during training, structured into mobility through policy and professional mediation, and feeds back into the domestic system through depletion of mentorship, wellbeing, managerial capacity, and care equity, thereby shaping the next cohort's migration intent.

What these accounts miss is that the benefits are conditional, unevenly distributed, and often weakly institutionalised. Remittances may sustain households while doing little to rebuild public care capacity. Return migration may remain aspirational, delayed, or professionally under-absorbed. Bilateral agreements may promise reciprocity while in practice prioritising destination-country shortages over source-country sustainability (47). Most importantly, the conventional debate treats migration as an event that follows a decision. The Nepal case assembled here suggests a different chronology and a different politics. Mobility should be understood as an aspiration–mediation–depletion cycle (Figure 1). Aspiration is formed during training as students encounter the gap between professional ideals and domestic realities. Mediation occurs when governance arrangements—recruitment policies, regulatory pathways, protected titles, and globally portable definitions of nursing—convert desire into a feasible exit. Depletion follows as the departure of experienced nurses weakens mentorship, strains managers, deepens inequity, and worsens the environments in which subsequent cohorts are trained. That depleted environment then helps generate the next round of aspiration.

This reframing also clarifies why migration governance cannot be treated as external to migration intention. Evidence from Nigeria shows that formal and informal rules, rights-based norms, workforce policy, and the perceived benefits of staying—all directly shape skilled health workers' migration intentions (73). The policy problem isn't persuading nurses to stay; it is how to interrupt the feedback loop through which professional undervaluation, structured mobility, and system depletion continually reproduce one another.

## 8 Policy options and the recommended pathway

If brain drain in Nepal is understood as an *aspiration–mediation–depletion* cycle, then neither patriotic appeals nor tighter moral scrutiny of nurses will solve it. The policy task is to make remaining in Nepal a professionally credible option, to prevent student formation from being damaged by system fragility, and to ensure that international mobility—where it occurs—returns some measurable value to the source system. The recommended pathway, therefore, is not prohibition but governed reciprocity: Revalue nursing domestically, safeguard the educational pipeline, transform one-way recruitment into capacity-building mobility, and adopt global professional standards in ways that strengthen rather than weaken local systems.

### 8.1 Domestic revaluation

Nepal cannot sustain a nursing workforce if the profession continues to be positioned as indispensable in rhetoric but marginal in pay, authority, safety, and advancement. A realistic reform package should begin with a national nursing workforce compact, jointly led by government, regulators, public and private employers, and nursing leadership. Its immediate deliverables should include a phased public/private-sector pay review, a protected career ladder from novice to specialist, educator, and managerial roles, and a “national workplace safety standard” covering violence prevention, reporting protections, and minimum supervisory expectations.

Retention policies should also be designed to address geographic barriers. Rural service cannot continue to depend on professional sacrifice alone. Hardship allowances, housing support, funded continuing education, and guaranteed priority for postgraduate training after a defined period of rural service are more likely to retain nurses than generic calls to serve the nation. Just as importantly, nurses must be given a real voice in governance. Every major hospital should include senior nursing representation in workforce planning, quality committees, and budget discussions. The nursing profession will continue to face attrition challenges as long as nurses are tasked with managing clinical risks without the necessary decision-making authority.

### 8.2 Protect the student-to-workforce pipeline

The second priority is to repair the educational pipeline. Student attrition is not the only concern; a more insidious problem is the production of graduates whose training has been clinically challenging, ethically disorienting, and psychologically exhausting. This requires moving from passive placement models to protected learning time. Clinical sites should be funded to appoint designated preceptors, with explicit workload credit for supervision rather than the current expectation that teaching will occur informally amid a crisis. Student-to-preceptor ratios should be set nationally and monitored during accreditation.

A structured graduate transition year is equally important. New graduates should not be used as a shock absorber for a shortage. Although Nepal does have a formal national transition-to-practice year for newly qualified nurses, workable prototypes exist in comparable LMIC settings. In South Africa, newly qualified nurses undertake a compulsory 12-month community service year before full professional registration, which has been described as a structured period for improving clinical skills, professional behaviour, and critical thinking, although the quality of implementation varies with mentorship and organisational support (74, 75). A related example comes from Ghana, where new graduate nurses' experiences in a one-year clinical rotation programme have been examined as part of workforce retention and transition support, further demonstrating that a supervised early-career consolidation year is feasible in resource-constrained settings (76). A supervised first year with progressive responsibility, scheduled debriefings, and protected mentorship would strengthen both competence and retention. In Nepal, such a model could be piloted first in high-volume teaching hospitals and provincial referral centres, where preceptorship capacity already exists in partial form, before being scaled nationally through accreditation-linked funding and workforce planning mechanisms. Nursing schools should also establish independent wellbeing pathways that sit outside formal academic assessment, so students can seek support without fearing that disclosure will damage their standing. Simulation and digital learning tools, such as gen AI, should be expanded, but as supplements to relational teaching rather than as substitutes for the human supervision they lack. When effectively implemented, these tools can alleviate the burden on overstretched facilities; however, when misused, they risk masking the detrimental effects of inadequate institutional support.

### 8.3 Replace one-way recruitment with reciprocal mobility governance

The third priority is to redesign bilateral mobility. Nepal should not approach international recruitment agreements as symbolic evidence of prestige, but as instruments that must generate enforceable returns. Any future MoUs should include four non-negotiable elements: *first*, destination-country financial contribution to domestic training capacity, including faculty development and clinical education infrastructure; *second*, transparent workforce dashboards linking recruitment approvals to current vacancy and training data; *third*, return or knowledge-transfer mechanisms, such as funded re-entry posts, visiting educator fellowships, or remote mentoring opportunities; and *fourth*, protections against deskilling, contract substitution, and discriminatory hiring practices in destination countries.

This would not eliminate migration; however, it would change the terms on which it occurs. The goal is to move from extraction to reciprocity. If a destination country benefits from a source country's trained nurses, it should share the costs of reproducing the workforce. *Mobility governance* should therefore be judged not by the number of nurses migrated, but by whether domestic training, supervision, and service capacity improved as a result.

### 8.4 Adopt the ICN definitions contextually, not mechanically

The fourth priority is to translate the renewed ICN definitions into Nepal's system without uncritically importing their risks. The definitions offer Nepal an opportunity to strengthen title protection, licensure, leadership claims, and public understanding of nursing as a science-based and rights-grounded profession. But that opportunity will be lost if professional elevation occurs without financing, bridging pathways, and inclusion.

A national implementation task force should therefore map existing laws, curricula, and regulatory structures against the ICN definitions and identify what can be adopted immediately, what requires phased financing, and what must be adapted to Nepal's nursing cadre mix. Protected-title reform should be accompanied by bridging routes for auxiliary and community-based providers, so professional consolidation does not unintentionally weaken frontline service delivery.

## 9 Conclusion

Nepal is not an outlier in the global nursing crisis; rather, it serves as a critical case study of how professional undervaluation, state-sanctioned recruitment, and transnational norms intersect to fundamentally restructure the workforce. What is routinely framed as a story of individual mobility is actually a broader political narrative about how nursing is financed, regulated, and extracted across deeply unequal health systems. In Nepal, the fallout extends far beyond vacancy statistics; it is visible in compromised clinical learning, ethical strain among students, moral distress among nursing leadership, and compounded inequities for historically underserved populations.

Ultimately, this analysis demonstrates that workforce out-migration cannot be treated simply as a retention problem. The paradigm of retention is conceptually flawed because it assumes a linear process in which nurses are successfully produced and subsequently lost. As the Nepalese context reveals, the crisis is far more severe: the profession's systemic reproduction is being actively undermined. When students are trained in unsupportive clinical environments, when experienced nurses migrate without reciprocal reinvestment, and when bilateral policies prioritise labour portability over domestic capacity, the system effectively consumes the conditions for its own renewal. So-called “*brain drain*” is therefore not an inevitability of globalisation, but a profound governance failure in the creation, sustenance, and valuation of nursing itself.
